# Annotations

**Published:** 1906-01-06

**Authors:** 


### ANNOTATIONS.

### Mr. Edgar Speyer's Prize.

It will be remembered that in the early part of last year Mr. Edgar Speyer offered a prize of £100 and a silver cup for the best essay on the economical management of an efficient voluntary hospital. The competition was open to the paid secretaries and assistant secretaries of all voluntary hospitals in England, Scotland, and Ireland. The announcement is now made that the prize has been awarded to Mr. Godfrey H. Hamilton, Secretary of the National Hospital for the Paralysed and Epileptic. The judges who assisted Mr. Speyer in the decision report that the essay sent in under the motto "Dum Spiro, Spero" was nearly equal in merit to the successful essay, and Mr. Speyer is desirous of hearing from the writer with a view to its publication. One of the conditions laid down when Mr. Speyer's offer was made public was to the effect that the successful competitor must consent to the publication of his essay, free of expense to himself, under his own name. We demonstrated at the time how embarrassing this condition might prove to be, and how it might to some extent limit the scope of the competition—having regard to the criticisms which a man of honesty and discernment must make when writing on hospital economy. Although our suggestion that the condition should be waived did not meet with approval, it seems as though the fears we expressed are about to receive justification. The competitor who is now being sought for may, it is true, have omitted to enclose his name with his essay by inadvertence, but we should not be at all surprised to learn that he has purposely followed this course in obedience to the dictates of worldly wisdom. In the meantime we offer our sincere congratulations to Mr. Hamilton for the distinction which Mr. Speyer's thoughtful generosity has given him the opportunity to gain.

### Quackery.

There has been formed in Germany a society for the combating of quackery, and it is proposed that we should follow suit. Certainly all thinking people must be agreed that the utterly unscrupulous ways of the advertising quack should be brought within the purview of the law, but it is not necessary to labour the difficulties of converting this pious resolution into practice. Several factors combine to render the profession of the quack (if we may be pardoned the paradox) as profitable as it is difficult of assault, but the root-cause is the failure of the medical profession to cure every variety of morbid process. So long as there remains a degeneration which is not amenable to treatment, so long as there remains a functional distress which keeps its victim melancholy company at intervals throughout a long life, so long will humanity reach out into the unknown domains of quackery for the relief which legitimate medicine has failed to render. Consequently public opinion is against us, all the more that its charity is not proof against the suspicion that we of the profession are not free, in our strictures upon quacks, from an undercurrent of jealousy and self-seeking. This attitude of the public should not prevent us, however, from doing what we can to scotch an evil of great dimensions. It is hard to read without uncommon indignation advertisements (in reputable papers) which describe in glowing detail a multitude of minor and trifling symptoms, and end by warning the unhappy reader that any one of these is an index of oncoming paralysis. But the people love to have it so, and no society will change their opinion easily. None the less there is room for hope that with the growth of knowledge a wiser spirit will pervade our countrymen. In that millennium perhaps it will at last be more profitable to practise legitimate medicine than to prescribe and name a pill and persuade a syndicate to finance the puffing of it.

### Vaccination Officers and Guardians' Interference.

Recently the Guardians of the Blaby Union appointed a vaccination officer on condition that he agreed to refrain from any legal proceedings against defaulters without the sanction of the Guardians. But a vaccination officer, though he is appointed by the Guardians of the Union where he works, must have the appointment sanctioned by the Local Government Board. That body, having been duly informed of the appointment, have replied to the effect that the condition attached to the election makes it invalid. The Secretary of the Local Government Board says in his letter: "The duties of the vaccination officer in relation to proceedings against defaulters as regards vaccination are determined by the Vaccination Acts, and cannot be curtailed or restricted by any resolution of the Guardians, and any resolution of the Guardians intended to have any such effects would be *ultra vires*." The Guardians had also stated that they would decline to pay any costs or charges for prosecutions undertaken without their sanction, and on this point they are informed that they cannot by any resolution they may pass relieve themselves of their duty to comply with the regulations of the Acts. It would be a good thing if this letter were sent not only to the Blaby Guardians, but to every Board of Guardians in England. As a rule there is very little public interest in the election of Guardians, and it is easy for anti-vaccinators to obtain election to a position which they often use almost exclusively for the purpose of defeating the provisions of the Vaccination Acts. On the other hand, it would be well if there were some clearer definition of what it means to be a conscientious objector. Some magistrates will grant a certificate of exemption on a statement which another will put aside as insufficient. If a man has applied for an exemption certificate, and, having been refused, declines to have the child vaccinated, he very naturally resents being treated as if his default was the result of mere carelessness. Since the law provides for conscientious objectors, it seems to us that those who take the trouble to apply for a certificate of exemption within the prescribed time should obtain it without trouble, and there could then be no objection to the prosecution of those who had been too lazy or careless to make the necessary application.

